# Direct contacts of microglia on myelin sheath and Ranvier’s node in the corpus callosum in rats

**DOI:** 10.7555/JBR.32.20180019

**Published:** 2018-04-11

**Authors:** Jingdong Zhang, Xinglong Yang, You Zhou, Howard Fox, Huangui Xiong

**Affiliations:** 1. Department of Pharmacology and Experimental Neuroscience, University of Nebraska Medical Center, Omaha, NE 68198, USA; 2. Department of Clinical and Scientific Training, Affiliated Hospital to Academy of Military Medicine Sciences, Beijing 100071, China; 3. Center for Biotechnology, University of Nebraska at Lincoln, School of Veterinary Medicine and Biomedical Sciences, Lincoln, NE 68588, USA.

**Keywords:** Ranvier’s node, myelin sheath, microglia, contact, matured white matter tract

## Abstract

Over the recent years, it has been found that microglia pseudopodia contact synapses, detect sick ones and prune them, even in adult animals. Myelinated nerves also carry out plasticity in which microglia remove myelin debris by phagocytosis. However, it remains unknown whether microglia explore structures on nerve fibers, such as Ranvier’s node (RN) or myelin sheath, before they become debris. By double or triple staining RNs or myelin sheathes and microglia in healthy rat corpus callosum, this study unveiled direct contacts of microglia pseudopodia with RNs and with para- and inter-nodal myelin sheathes, which was then verified by electron microscopic observations. Our data indicated that microglia also explore unmyelinated nerve fibers. Furthermore, we used the animals with matured white matter; therefore, microglia may be actively involved in plasticity of matured white matter tracts as it does for synapse pruning, instead of only passively phagocytize myelin debris.

## Introduction

Microglia, the brain resident cells that can sense pathological tissue alterations, are believed to be quiescent in physiological circumstances^[1^–^2]^. However, in recent decades, microglia are found to be more than passive responders. They show high levels of activity with their pseudopodia exploring the surrounding environment, which is known as “dynamic surveillance of brain parenchyma”^[3^–^5]^. One of the newly observed physiological functions of microglia is their involvement in synaptic stripping or pruning^[4^–^5]^. Under a two-photon live-image microscope, fine microglia pseudopodia are viewed to extend to pre- and post-synaptic structures at a certain rate. Further, direct contacts of labeled microglia processes onto synapses and/or engulfed post-synaptic protein in microglia cytoplasm have been envisioned under the electron microscope^[4^–^6]^. During development, microglia are responsible for removing the less-used synapses^[5^,^7]^. This was verified by comparing microglia activity in neonatal visual cortex with normal visual experience to that activity in the visual cortex with monocular deprived visual experience^[8]^. In addition, possible participation of microglia in shaping white matter tracts in visual callosum has been reported in a similarly designed study^[9]^.

Like synapses in the neuropil, nerve fibers, especially the myelinated nerve fibers, also undergo plasticity in both developing and matured white matter tracts^[10^–^11]^. It is rational to contemplate that microglia may also surveil and maintain the integrity of white matter tracts. Myelinated nerve fibers among white matter tracts play a pivotal role in the brain communication between neurons or nuclei in a manner of saltatory conduction^[12]^. The Ranvier’s node (RN) and the internodal myelin sheath are key structures for the generation of saltatory conduction because they concentrate voltage gated sodium (Na^+^v) channels on the node and insulate internode axons with myelin sheath^[13^–^14]^. The node area is protected by microvilli of Schwan cells in myelinated peripheral nerve fibers as this area is extremely important for the rapid transmission of neuronal signals^[15^–^16]^. In addition, microvilli may be involved in stabilizing Na^+^v channels at the node through trans interaction of microvilli dystroglycan with nodal axolemmal molecules, since selective knockout of Schwan cell’s dystroglycan can result in nodal Na^+^v channel density reduction and nerve conduction slowdown^[16]^.

However, which structure in the brain white matter tracts can play the same role as microvilli does in peripheral nerves is still not clear. Fine process of astrocyte has been known to contact the RNs in the central nerve system (CNS), but the functional profile of these contacts are still equivocal^[17^–^18]^. A function proposed by some scholars is that astrocyte nodal processes buffer fluctuation of ions around the node, but the molecular details and the mechanism are still unknown^[17^–^18]^. Moreover, electron microscopy studies on contacts of labeled astrocyte processes with RNs in the corpus callosum (CC) and optic nerve have revealed that a considerable number of RNs therein are still uncovered or only partially covered^[17^,^19]^, leaving a broad room on nodal axolemma for other cellular components to reach. In light of the recent findings aforementioned, we hypothesized that microglia pseudopodium might extend to both CNS RN and myelin sheath, during developing myelination or post white matter tract maturation. Of particular interest was the role of pseudopodia in adult white matter tracts, as that might be related to some white matter injuries in adulthood. The concentration of Na^+^v channels at the nodal area^[13]^ allowed us to apply double and triple immunofluorescent staining of the node and microglia. In combination of labeling para- or inter-nodal domains by pertained markers, we showed in this work the potential contacts of microglia pseudopodia with the RNs and with para- and inter-nodal myelin sheathes in the rat CC. Finally, electron microscopy was conducted to verify contacts between microglia pseudopodium and RN. All experiments were performed using young adult rats of 45–55 days, when major developing myelination finished and white matter had developed into a matured stage^[20^–^21]^.

## Materials and methods

### Animals

Thirteen adult Sprague–Dawley rats (45–55 days old; 175–250 g; 7 male and 6 female), purchased from Charles River Laboratories (Wilmington, MA), were used in this study. Samples from ten rats (5 male, 5 female) underwent double and triple labeling, and then were observed under confocal microscopy. Samples from three animals (2 male, 1 female) were used for electron microscopy. All experimental protocols and animal care were carried out in accordance with the National Institutes of Health *Guide for the Care ofLaboratory Animals in Research* and approved by the Institutional Animal Care and Use Committee of the University of Nebraska Medical Center. All efforts were made to minimize animal suffering and the number of animals used in this study.

### Immunofluorescent staining for confocal microscopy

#### Double labeling of myelin sheath and microglia

Animals were euthanized with isoflurane and transcardially perfused with saline followed by 4% paraformaldehyde in phosphate buffer (PB; 0.1 mol/L, pH 7.2–7.4). The brain containing the CC was cut into 10-μm coronal or sagittal frozen sections and directly mounted on *plus-*coated slides. The sections with the CC were incubated with either rabbit anti-myelin basic protein (MBP; 1:200–1:500, Abcam, Cambridge, MA) plus goat anti- Ionized calcium binding adaptor-1 (Iba-1) or rat anti-MBP (1:100, Millipore, Temecula CA) with rabbit anti-Iba-1 (1:500, Wako USA, Richmond, VA) overnight at room temperature (RT). Alex Flour conjugated secondary antibodies (all diluted as 1:200–1:500; Molecular Probes, Eugene, OR) against primary antibodies were applied to display fluorescent labeling. Control staining for different antigens was carried out without primary antibody.

#### Double labeling of RN and microglia

Brain tissues were processed in the same way as aforementioned. The coronal sections were incubated with either rabbit anti-pan Voltage-Gated sodium channels (Pan Na^+^v; 1:200, Alomone, Jerusalem, Israel) with goat anti-Iba-1 (1:200–1:500, Abcam), or mouse anti-Na^+^v subunit 1.6 (Clone K87A/10; 1:100–1:200, UC Davis/NINDS/NIH NeuroMab, Davis CA) with rabbit anti-Iba-1 (1:300–1:500, Wako USA) for revealing RN and microglia with ramified pseudopodia. Alex Flour conjugated secondary antibodies (all diluted as 1:200–1:500; Molecular Probes) against primary antibodies were applied to display fluorescent labeling.

#### Triple labeling of RN, paranodal/internodal myelin sheath and microglia

Brain tissues were processed in the same way as aforementioned except that 7.5% saturated picric acid (75%) was added to the fixative. The CC sections were immunostained with a cocktail of rabbit anti-MBP (1:300, Abcam), mouse anti-Na^+^v 1.6 (1:200, UC Davis NeuroMeb) and goat anti-Iba-1 (1:200, Abcam). Then, Alexa Flour 488 conjugated donkey anti-rabbit, Alexa Flour 568 attached donkey anti-mouse and ByLight 405 linked donkey anti goat (1:200; kindly provided by Microscopy Facility at University of Nebraska at Lincoln) were applied to visualize the structures labeled by primary antibodies.

#### Triple labeling of RN, paranodal domain and microglia

Brain tissues were processed in the same way as aforementioned. As has been known, a characteristic of the RN in myelinated nerve fiber is that the node is always nested in-between two paranodal domains that are contactin-associated protein 1 (Caspr1) positive^[22]^. Thus, antibody against Caspr1 was added in triple labeling of RNs, paranodal domains and microglia. Then, the sections were incubated in cockatiel of rabbit anti-pan Na^+^v (1:300, Alomone), mouse anti-Caspr1 (1:200, UC Davis NeuroMeb) and goat anti-Iba-1 (1:200, Abcam) overnight at RT. Next, Alexa Flour 568 conjugated donkey anti-rabbit, fluorescein conjugated donkey anti-mouse and ByLight 405 conjugated donkey anti goat (provided by UNL) were applied to reveal the node, paranodal domain and microglia.

### Preparation of tissues for electron microscopy

#### Immunohistochemistry staining

Animals were euthanized by isoflurane and transcardially perfused with saline followed by PB, as mentioned above, containing 2% paraformaldehyde and 0.5% glutaraldehyde with 7.5% saturated picric acid (75%). The front brain containing the CC was extracted and cut into 50-μm coronal sections by a vibratome and saved in PB. The sections were immunohistochemically stained as previously described^[23]^.

#### Osmification and plate embedding

The sections then underwent osmification and plate embedding as described previously^[23]^. The labeled microglia in the embedded sections were identified under light microscope (*Fig. 4A*–*B*), and then trimmed off for ultrathin sectioning. Ultrathin sections were cut with Leica EM UC7 Ultracut microtome, stained with lead citrate and observed with Hitachi H7500 TEM (at Core Research facility, Center of Biotechnology, University of Nebraska at Lincoln). And the work continued afterward at Computer Imaging and Microscopy Core Facility, Affiliated Hospital of Academy of Military Medicine Sciences, Beijing, China. Ultrathin sections were cut with LKB ultra-microtome, stained with lead citrate and observed with FEI Tecnai G^2^ Spirit transmission electron microscope.

### Imaging acquisition and processing

Digital images of fluorescent labeling were collected using Nikon A1 confocal system (Japan) equipped with Nikon 90i upright fluorescence microscope and BioRad Laser Sharp 2000 imaging program (Digital BioRad Center, Pleasanton, CA, USA). Alexa Fluor 568 labeling was viewed through 561 excitation laser line with 10 nm resolution of spectra. Alexa Fluor 488 and ByLight 405 were viewed through 488 and 405 excitation laser line, also through 10 nm resolution spectra. Confocal images were captured with ×20 and ×40 objective at iris of 2.0–2.5 in box size of 1,024×1,024. All Z-scan was set up at 1 μm layer of laser scan step and saved as “avi” video files and the clearest double or triple labeling image was selected and converted to Photoshop 7.0.1 (Adobe, CA, USA) through Bio-Rad Plug-In software and stored as “tiff” file at 1,024×1,024 pixels. The electron microscopy microphotographs presented here were taken with FEI Tecnai G^2^ Spirit electron microscope.

## Results

### Double labeling of myelin sheath and microglia

**Fig.1:**
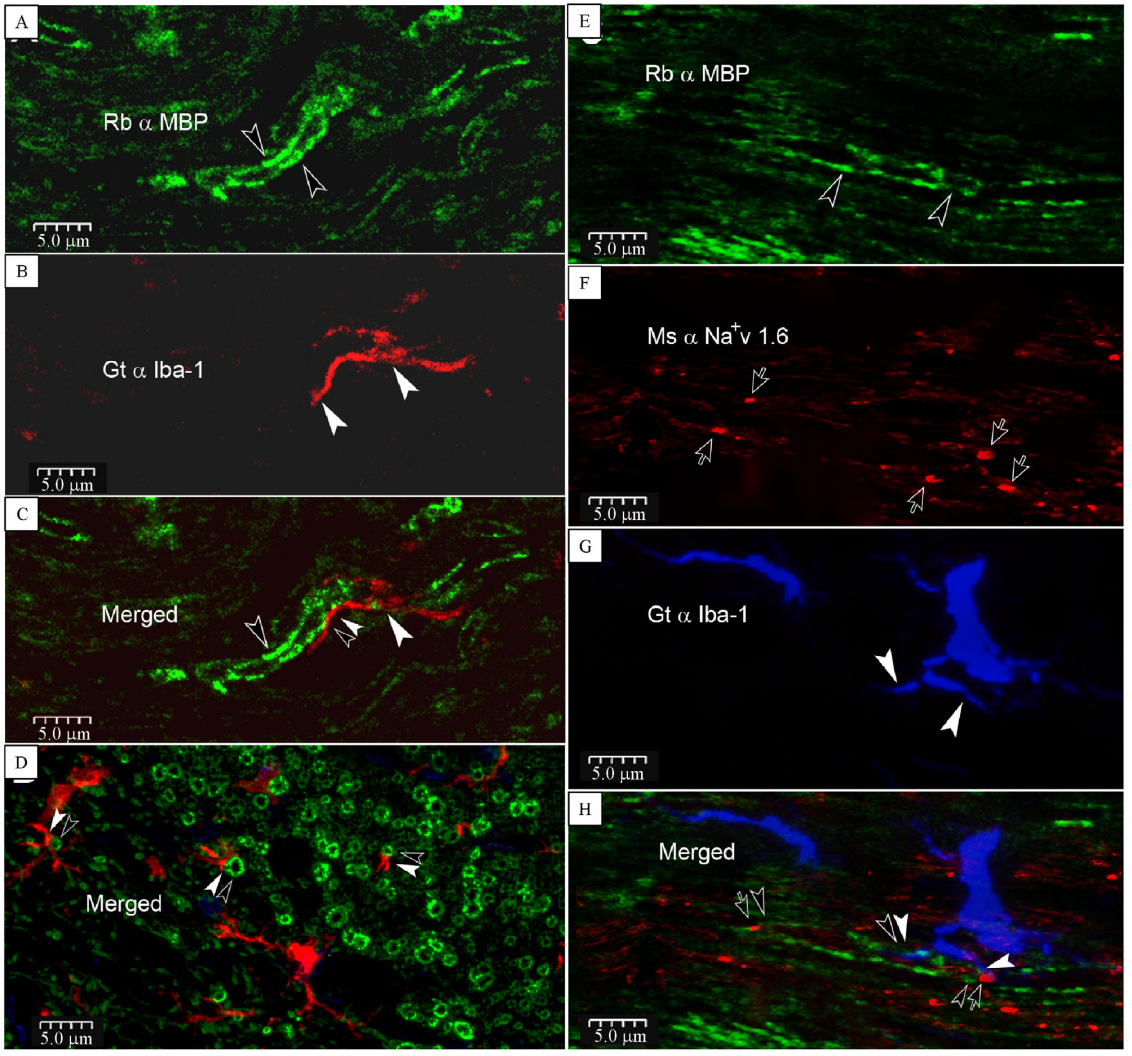
Direct contacts of microglia pseudopodia with inter-, para-nodal myelin sheath and probably Ranvier’s node (RN). A¨CC: Iba-1 positive microglia pseudopodium (arrowheads) directly contacts onto myelin basic protein (MBP) labeled myelin sheath (opened arrowheads) in the corpus callosum (CC, paired arrowheads). D: contacts (paired arrowheads) between microglia pseudopodia and myelin sheathes viewed in cross section. E¨CH: a single microglia with its pseudopodia (arrowheads) in close apposition upon a RN (opened arrows) and its para- and internodal myelin sheath (opened arrowheads). Merged panel shows Iba-1 positive process contacts on a MBP labeled internodal (paired down-point arrowheads) myelin sheath. Meanwhile, the other pseudopodium seems to contact with a RN and its paranodal apparatus (upper-point opened arrow and arrowhead, plus a left-point arrowhead). This image is constructed from 3 scanned layers to show an entire single microglia with both soma and processes.

Using MBP to label myelin sheath and Iba-1 to mark microglia, we observed in both coronal (*Fig. 1A–C*) and sagittal sections (*Fig. 1B*) that microglia pseudopodia (arrowheads) explore the inter-nodal myelin sheath (opened arrowheads) frequently (paired arrowheads and opened arrowheads in *Fig. 1C* and *D*). All control staining without primary antibody resulted no labeling.

### Triple labeling of RN, para-/inter-nodal myelin sheath and microglia

Unlike Caspr1, which is expressed only on paranodal domains, MBP can mark both paranodal and internodal myelin sheath. We encountered a paranode – RN – microglia complex, characterized by MBP positive paranodal apparatus immediate abut against a Na^+^v 1.6 labeled RN (opened arrows) that was touched by a microglia process marked by Iba-1 (*Fig. 1E–H*). *Fig. 1H* showed that a single microglia with its pseudopodia (arrowhead) appeared to explore a RN and its nearby paranodal (a pair of opened arrows and arrowhead pointing up), and a continued internodal (a pair of arrowheads and opened arrowhead pointing down) myelin sheath simultaneously.

### Double labeling of RN and microglia

**Fig.2:**
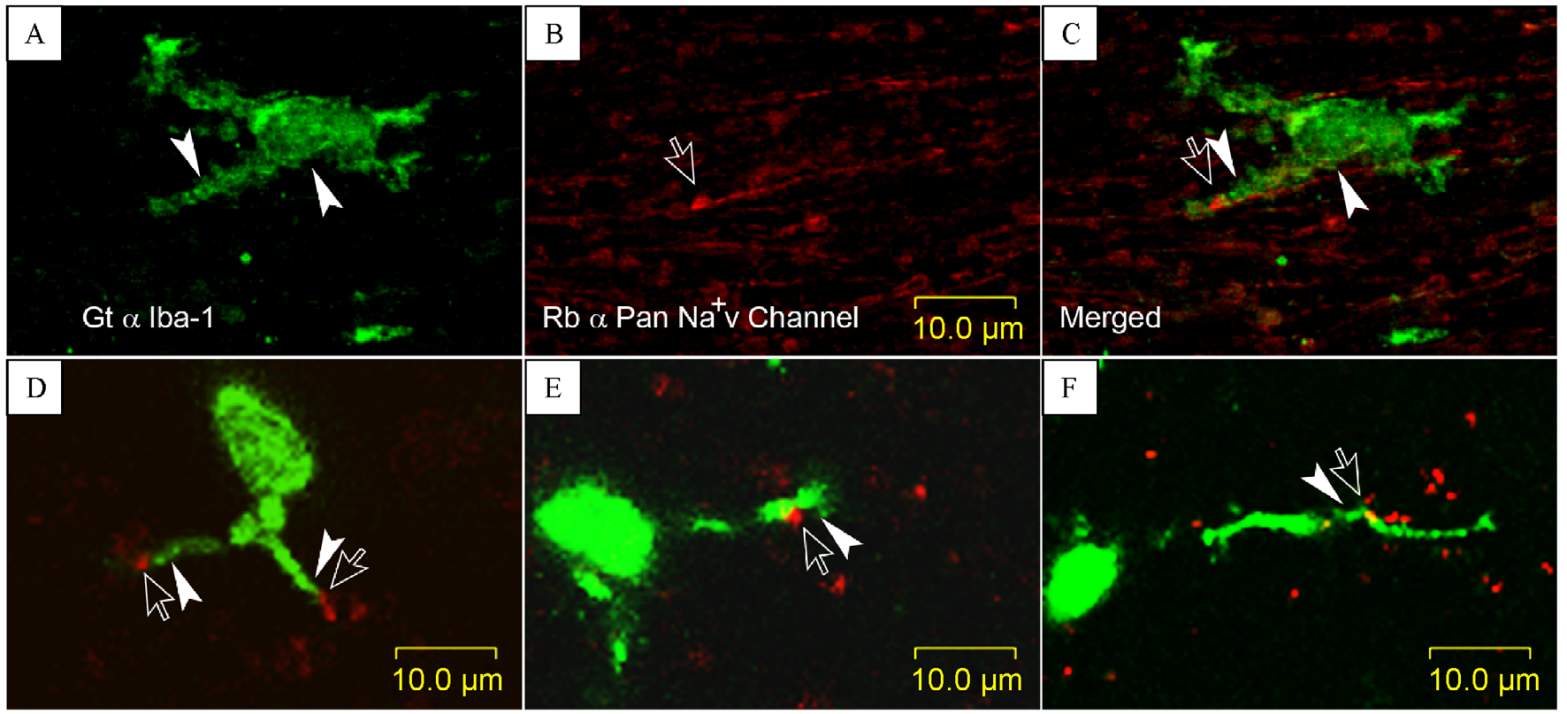
Direct contacts of microglia pseudopodia with RNs or Na^+^v channel clusters. A¨CC: a microglia with its pseudopodium (arrowheads) immediately contacts a Na+v channel cluster (opened arrow) that appears to be lining with an unmyelinated fiber (paired down-point arrow and arrowhead in Merged). D¨CF: microglia pseudopodia contact onto RNs or RN like Na+v channel clusters (paired opened arrow and filled arrowheads).

Under laser scan of 1-µm layer of the CC section, it was explicitly observed that microglia pseudopodia (arrowheads) immediately contact the possible RNs or clusters of Na^+^v channel (opened arrows in *Fig. 2*). However, in *Fig. 2A–C*, a nerve fiber lining and connecting with the labeled node (opened arrow) was also stained by anti- Na^+^v antibody, inferring this nerve fiber was an unmyelinated fiber, since the Na^+^v channels appears to evenly distribute along the unmyelinated nerve fibers in addition to aggregating in the node-like area^[24]^. Besides, clusters of Na^+^v channels on unmyelinated nerve fiber is suggested by a recent finding that Na^+^v channels piled fragmentally upon lipid rafts on axolemma of node like area in unmyelinated fibers^[25]^.

### Triple labeling of RN, paranodal domains and microglia

**Fig.3:**
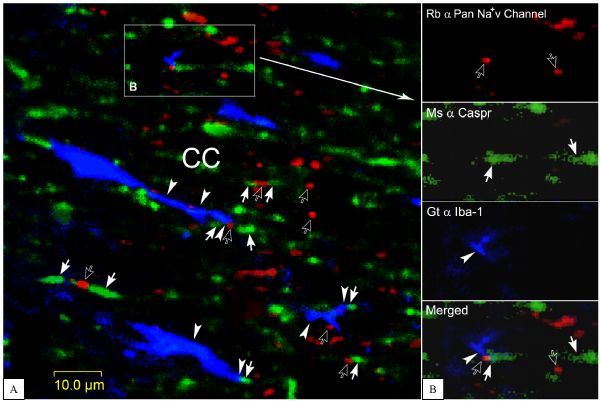
Verification of microglia pseudopodia contact with RNs and paranodal domains. A: three typical structures are viewed herein. 1, Na+v channel positive RNs (opened arrows) nested in-between of Caspr 1 labeled paranodal domains (arrows and an opened arrow together); 2, microglia pseudopodia (arrowheads) contact on RNs lining with paranodal domains (arrow, arrowhead and opened arrow together); 3, microglia pseudopodia are tightly apposite upon paranodal domains (arrowhead and arrow together). B: from a framed area in A, showing a labeled microglia pseudopodium immediately contacts a RN and its paranodal domain (arrowhead, opened arrow and arrow together in merged panel). This image was constructed from 3 laser scanned layers. CC, corpus callosum.

As the RN in myelinated nerve fiber is always situated in-between of paranodal domains that express Caspr1^[22]^, we attempted to show the node and paranodal domain simultaneously to clarify whether microglia pseudopodia indeed explore the RN in myelinated nerve fibers. Then, by means of triple labeling, we unveiled that microglia pseudopodia (arrowheads) stretched to contact RNs labeled by Na^+^v channels antibody (opened arrows) and/or paranodal domains marked by Caspr 1 (arrows in *Fig. 3A*). In some cases, the Iba-1 positive microglia pseudopodia are closely apposite upon both RN and paranodal domains simultaneously (*Fig. 3B*, arrowhead, opened and filled arrow together in Merged), although we did not know whether these contacts were instant or constant.

### Electron microscopic observations

**Fig.4:**
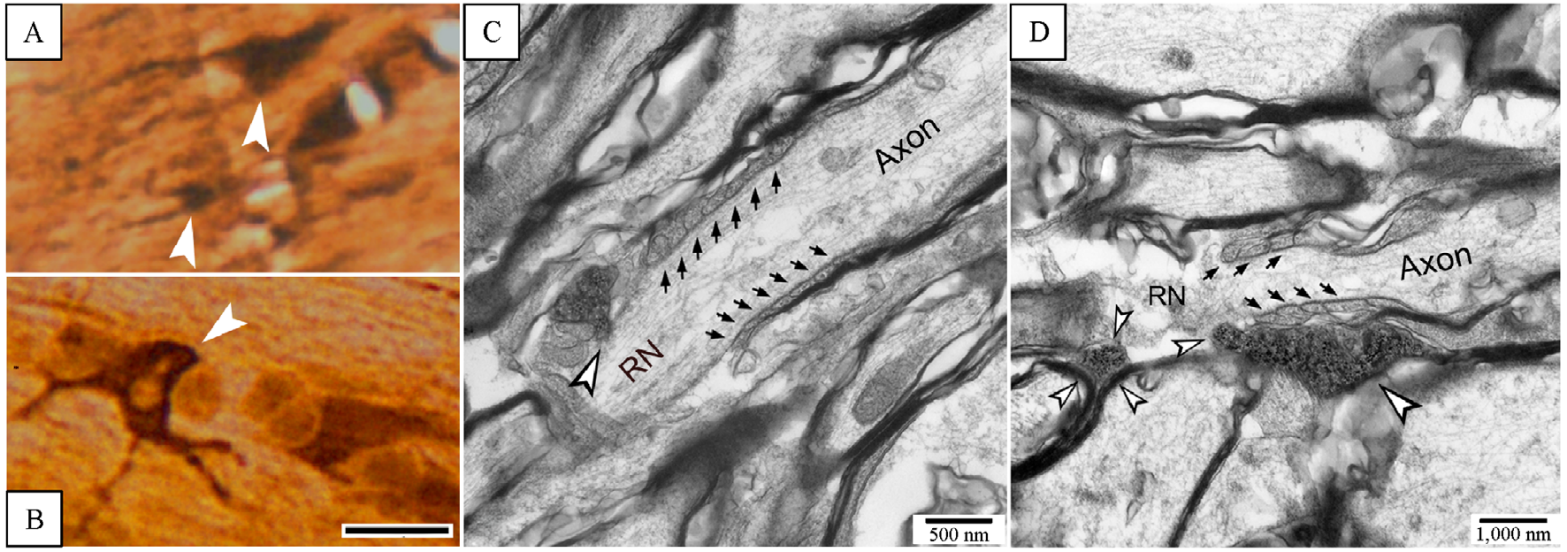
Ultrastructural view of microglia pseudopodia contacts on RNs. A and B: Iba-1 immunohistochemically labeled, osmicated and plate-embedded sections observed under bright-field microscope, on which the labeled microglia and pseudopodia (arrowheads) could be seen clearly. Scale bar = 15 ¦̭. C and D: ultrathin sections cut from A or B, exhibiting ends of microglia pseudopodia (opened arrowheads) direct contact on naked axolemma of RN area. Apposition of microglia pseudopodium upon paranodal myelin sheath is also viewed (D), but there seems to be a tiny gap (about 5¨C10 nm) between them. Aligned arrows point towards spiral loops of paranodal apparatus.

With plate-embedding, labeled microglia and their pseudopodia could be visualized under conventional light microscope (*Fig. 4A* and *B*, arrowheads). The tissue block containing labeled microglia (*Fig. 4A*, *B*) was marked and extracted from plate-embedded sections under anatomical microscope for further preparation of ultrathin section. Ultrathin sections were cut along with longitudinal axis of nerve fibers in the CC as shown in *Fig. 4C* and *D*. Ends of microglia pseudopodia (opened arrowheads in *C* and *D*) were seen to contact axolemma on RNs. These contacts appeared between the naked axolemma on the node and the ends of pseudopodia without any gap (*C *and *D*). The contact between paranodal myelin sheath and microglia pseudopodia was also observed (*Fig. 4D*). That paranodal domain was termed as axoglial apparatus ultrastructurally characterized by spiral loops formed by paranodal myelin sheath (pointed by aligned small arrows in *C* and *D*). There appeared to be a tiny gap (about 5–10 nm) between plasm membrane of microglia and paranodal myelin sheath; however, the other end of pseudopodia seemed to have direct contact with the RN and another two myelin sheathes of myelinated fibers (*Fig. 4D*, opened arrowheads).

## Discussion

To the best of our knowledge, the findings presented here, demonstrate for the first time that microglia pseudopodia directly contact with RN and paranodal or internodal myelin sheath in healthy young adult CNS white matter. It has been reported that in rodents, the maturation of white matter tracts in the CC is between 40 **–**45 postnatal days^[20^ **–**^21]^ when about 30% of axons have been myelinated and nerve fiber compound action potentials become stable^[20^ **–**^21]^. Therefore, those direct contacts of microglia pseudopodia upon RNs and myelin sheath are probably associated with plasticity of myelinated nerve fibers^[10^ **–**^11]^ in adulthood rather than developing myelination during peak myelination. On the other hand, there may be contacts between microglia pseudopodia and clusters of Na^+^v channels on unmyelinated nerve fibers^[24^–^26]^, because about 70% of nerve fibers in matured CC are unmyelinated fibers^[20^ **–**^21]^. Considering that microglia dynamically survey the surrounding environment in grey matter^[3]^, it is not surprising that microglia would explore both RNs in myelinated fibers and node-like regions on unmyelinated nerves^[25^ **–**^26]^, playing a similar role as in the grey matter^[3]^. However, whether microglia play any special role for myelinated nerve fibers by close contact with RNs is not clear, but that should not be overlooked.

Ample evidences have shown that CNS white matter tracts persist on myelination and/or remyelination after the developing myelination period, and indeed, are likely to do so throughout the lifespan^[10^,^27]^. For example, further myelinating unmyelinated nerve fibers and/or remodeling or replacing established myelin sheath continue when organism’s task performance changes, *i.e.* a new skill-related function is established or *via* the aging process^[10^,^27]^. Physiological remodeling or replacement of myelin sheathes should not compromise their normal function, but old myelin sheaths need to be removed before new oligodendrocyte processes begin to wrap axons^[28^ **–**^29]^. Thus, the time window from the identification and breakdown of sick myelin, to the rebuilding of new sheath could not be too long. The direct contacts of microglia pseudopodia onto myelin sheath might provide more precise data about the condition of myelin sheathes. Microglia can actively remove myelin debris during demyelination; however, it remains unknown whether the microglia can recognize and remove sick or degrading myelin sheathes before they break into debris. It is also unclear whether those microglia pseudopodia are periodically or constantly contacting the node^[3]^, which could be further studied under two-photon live-image microscopy.

Furthermore, the RN is a key structure to accomplish saltatory conduction as aforementioned. It is rational to presume if the nerve fibers malfunction, activities of the RNs will be altered and *vice versa*. Thus, the best approach to monitor physiological states of a myelinated nerve fiber is to monitor its RN activity. The ratio of ions around a RN is absolutely important for the generation of saltatory conduction^[30]^; whereas, the concentration fluctuation of some key cations reflects the RNs functional states^[12^ **–**^13]^. It has been demonstrated that microglia sense density changes of some cations; for instance, the increment of extracellular potassium (K^+^) concentration can elicit microglia activation^[31]^. A few of studies uncovered that a reduction of extracellular Ca^2+^ concentration around neuronal somata or dendrites evoked the convergence of microglia pseudopodia which then moved toward dendrites^[32^ **–**^33]^. This finding suggests microglia may be able to sense Ca^2+^ surrounding the RNs. Commonly, Ca^2+^ around RN is stable and Ca^2+^ influx occurs only when axolemma or paranodal apparatus are disrupted^[34^ **–**^35]^. However, physiological Ca^2+^ influx through the RN axolemma was found recently in cases where the axons discharged at a remarkably high frequency^[36^ **–**^37]^. Hence, monitoring the K^+^ and/or Ca^2+^ concentration around RNs might be one of the duties for those microglia to have close apposition or contact upon the RNs.

If microglia monitor and maintain the function of RNs by means of close apposition of their pseudopodia upon RNs, it is intriguing for us to know what will happen when those processes are shortened or shrunken upon microglia activation. Our recent study showed that the activation of microglia *per se* would induce white matter tract function changes, especially declination of axon compound action potentials^[38]^. The length and thickness of microglia pseudopodia dramatically changed from delicate soma with thin ramified pseudopodia to amoeboid cells with short hyper-ramified morphology when microglia were activated. The number of contacts between microglia and RNs or myelin sheath might change in this circumstance. More interestingly, the extent of declination of compound action potentials is correlated with the states of microglia activation^[38]^. However, the functional significance of those contacts of microglia pseudopodia with RNs and para- or inter- nodal myelin sheathes is still an enigma. Nonetheless, this finding will open a new avenue for exploration into the effect of microglia, in addition to overt phagocytosis and/or invisible cytokines secretion, on white matter tract integrity.
